# Hermetic bags maintain soybean seed quality under high relative humidity environments

**DOI:** 10.1016/j.jspr.2022.101952

**Published:** 2022-03

**Authors:** Dieudonne Baributsa, Ibrahim B. Baoua

**Affiliations:** a Department of Entomology, Purdue University, West Lafayette, IN, 47907, USA; b Département des Sciences et Techniques de Productions Végétales, Université Dan Dicko Dankoulodo de Maradi, Maradi BP 465, Niger

**Keywords:** Smallholder farmers, Humid tropics, Seed storage, Postharvest loss, Airtight storage

## Abstract

Soybean seed quality is affected by high relative humidity (r.h.) during storage in the humid tropics resulting in loss of germination. This study assessed the effectiveness of hermetic bags in preserving soybean seed quality when stored at high r.h. over three months. Treatments consisted of Purdue Improved Crop Storage (PICS) and control polypropylene (PP) woven bags kept at 30 and 80% r.h. Moisture content and germination were measured each month. Moisture content did not change, except in seed stored in PP bag at 80% r.h. where it more than doubled after one month. There was no significant difference in germination rates between soybean seed stored in PICS bags at 30 and 80% r.h. over three months. Soybean seed stored in PP bag at 30% r.h. had germination rates similar to those observed in PICS bags at 30 and 80% r.h., except after the third month where it significantly decreased compared to PICS bags at 30%. Germination rates of soybean seed stored in PICS at 30% and 80%, and PP bags at 30% decreased by about 3, 6, and 7%, respectively. However, the germination rates of soybean seed stored in PP bags at 80% r.h. dropped by 98% after three months. There was a significant negative correlation of −80.6% (Pearson correlation) between moisture content and seed germination. Farmers and seed producers/traders in the humid tropics can safely preserve soybean seed using commercially available hermetic bags.

## Highlights

• PICS bags maintain soybean seed quality stored at 80% relative humidity (r.h.) for 3 months.
• Moisture content of soybean seed stored in PICS bags at 80% r.h. for 3 months remained the same.
• Moisture content of soybean seed stored in PP at 80% r.h. for 3 months increase by a factor of 2.27
• Germination of soybean seed stored in PICS and PP bags at 80% r.h. for 3 months were 93.33% and 0.67%, respectively.
• There was a significant negative correlation (−80.6%) between moisture content and germination of soybean seed.

## 1 Introduction

Soybean, *Glycine* max (L.) Merr., is gaining popularity in Sub-Sahara Africa and other regions of the world due to its economic potential to alleviate poverty and improve nutrition (Mahoussi et al., 2020). Although soybean is classified as an oilseed, it has a high protein content (Bilyeu et al., 2011). Soybean production is being expanded to meet the growing demand for animal feed (e.g., poultry), human consumption (e.g., soy milk), and industrial products (e.g., oil). In developing countries, soybean production is limited by access to and unavailability of quality seed (Chirchir et al., 2017; Mohammed et al., 2018). As a consequence, often farmers rely on low-quality seed from local markets or their stores (Sperling et al., 2020). Planting poor-quality seed can result in low production and economic losses (De Vitis et al., 2020; Shelar et al., 2008).

Farmers are faced with several challenges in preserving seed during storage including biotic (e.g., insects, fungi, and rodents) and abiotic factors (e.g., humidity, moisture, and temperature) (Ali et al., 2015, 2020; Ndimande et al., 1981). Soybean seed stored in the humid tropic is severely affected by ambient temperature and relative humidity (r.h.) (Chirchir et al., 2017; Ndimande et al., 1981). Soybean seed stored at high r.h. deteriorates faster due to accelerated physiological and pathological damages leading to loss of viability (Miah et al., 2006; Shelar et al., 2008). Due to the seed's hygroscopic nature, its moisture content changes in response to surrounding atmospheric r.h. affecting seed longevity, as well as seed germination (De Vitis et al., 2020; Nkang and Umoh, 1997).

Several methods have been assessed to preserve soybean seed including aluminum foil, polyethylene bags, raffia bags coated with polyethylene liners, laminated packaging, coating seed with polymer barriers, and cold storage (Ali et al., 2015, 2020; Tatipata, 2010; West et al., 1985). Some of these storage methods are effective but expensive, not practical, or commercially unavailable to smallholder farmers. Hermetic bags, that are used by smallholder farmers to store grain and seed, have the potential to preserve soybean seed as well. The Purdue Improved Crop Storage (PICS) technology, one of the hermetic bag brands, has been marketed in more than 35 countries in sub-Saharan Africa, Asia, Latin America, and the Caribbean (Baributsa and Ignacio, 2020). PICS bags are relatively affordable and very effective in preserving various crops (Murdock and Baributsa, 2014).

The PICS technology, composed of two liners fitted into a woven polypropylene (PP) bag, minimizes the change in relative humidity inside the bag by restricting the movement of air in and out of the bag (Bakoye et al., 2020; Williams et al., 2017). This helps to maintain grain and seed quality during storage. No studies have explored the use of PICS bags for soybean seed storage. This research assessed the quality of soybean seed stored in hermetic PICS bags at high relative humidity for three months. Results would be useful to smallholder farmers and seed producers/traders who often need to stored seed, and development partners who promote soybean to improve nutrition, food security, and income of farmers.

## 2 Materials and methods

### 2.1 Experimental setup

The experiment started on March 29, 2017, and ended on June 29, 2017. Soybean seed, Viking 2265 variety, was purchased from Johnny's Selected Seeds (Winslow, ME, USA) in January 2017. Viking 2265 is an organic seed variety that was not treated with chemicals. The experiment had four treatments of two types of bags (PICS and control PP woven bags) kept at two different relative humidity (30 or 80%). The control PP woven bags are commonly used by farmers to store their soybean grain and seed at prevailing ambient environmental conditions where r.h. can go beyond 85% (Ali et al., 2020). PICS bags are currently used by smallholder farmers around the world to primarily store grain. Storing soybean seed in PICS and PP woven bags at 30 or 80% r.h. would reflect smallholder farmers' conditions in both humid and non-humid tropics.

Smaller PICS and PP bags of 34.29 cm × 34.29 cm were made by cutting down individual 50 kg PICS bags into the appropriate size and sealing them using an electrical heat sealer (Uline H-86 Impulse Foot Sealer; Pleasant Prairie, WI, USA). PICS bags consisted of two liners fitted into a PP woven bag while the control consisted of a PP woven bag only. Each bag was filled with 1.5 kg of soybean seed and then each layer of the bag was tied shut with a plastic zip-tie. Each treatment had nine replicates (total of 36 bags) of which three were opened each month (destructive sampling) to assess seed quality. Once filled with soybeans, the bags were placed in one of the two Caron Insect Growth Chambers (Model 6025–1, 115 VAC, Caron Growth chambers, OH, USA) conditioned at 30% or 80% r.h. A total of 18 PICS and PP bags were randomly stored on three shelves inside each of the two Caron Insect Growth Chambers.

### 2.2 Data collection

The experiment was conducted for three months. Data were collected at the beginning of the experiment to establish the baseline and once each month until the end of the experiment. The following parameters were measured at the beginning, during, and at the end of the experiment:

***a) Oxygen consumption***: To allow oxygen measurements, a small hole was made in each PP bag before filling it with grain to accommodate a 4 cm diameter upper Petri dish plate which was sealed to the inside of the bag with a hot glue gun, covering the hole. On the inside of this Petri dish plate, an OxyDot was affixed so that the internal oxygen concentrations could be measured. For PICS bags, a 4 cm diameter upper Petri dish plate was sealed to the inside of the inner-most liner with a hot glue gun, before filling the bag with grain. A small hole of the size of the upper Petri dish plate was made in the outer woven bag of the PICS bag to ease the reading of oxygen. Oxygen was measured every day using an Oxysense 5250i oxygen reader device (Industrial Physics, Devens, MA) from the beginning to the end of the experiment.
***b) Temperature and relative humidity***: After each bag was filled with grain, a Lascar EL-USB-2 data logger (Lascar, Erie, PA, USA) was randomly put in one repetition of each treatment/opening frequency group. The data loggers collected temperature and relative humidity every 30 min for the duration of the experiment.
***c) Moisture content***: The moisture content of soybean seed was determined in each of the experimental units using a DICKEY-John mini-Gac moisture analyzer (Dickey-John, Auburn, IL, USA). The moisture content was measured at the beginning and each opening until the end of the experiment. Only one measurement was taken for each replication.
***d) Germination***: Germination rate was determined using the following method. From each opened bag 100 seeds were randomly collected and divided into four samples. Each sample of 25 seeds was put into a Petri dish lined with Whatman # 190 mm qualitative filter papers. These filter papers were sprayed with water once a day. Each petri dish was examined daily for germinated soybean seeds. A soybean seed was considered germinated once the emerged radicle was found to be 1–2 cm long. To facilitate ease of counting, germinated seeds were disposed of each day. To assess the vigor of soybean seeds, the germination index (GI) was calculated using the following formula: GI = Σ(Gt/Tt) where Gt is the number of seeds germinated on day t, and Tt is the number of days (Dehnavi et al., 2020).

### 2.3 Statistical analysis

The ANOVA test was used to compare means among the treatments for oxygen levels, moisture content, germination, and germination index. The Student–Newman–Keuls (SNK) was used to see which specific pairs of means were different. Analyzes were performed with SPSS 26 software.

## 3 Results

### 3.1 Oxygen levels inside bags

Oxygen levels among the treatments did not vary, ranging between 19.4 and 19.5% (v/v) (F = 0.24; df = 3; p = 0.88) at the start of the experiment to 19.5–19.7% (v/v) (F = 1.05; df = 3; p = 0.38) after 3 months of storage. No significant difference was observed in oxygen levels among treatments between the start and the end of the experiment (F = 2.89; df = 1; p = 0.89). No insect pests were observed on the soybean seed throughout the experiment.

### 3.2 Relative humidity inside bags and seed moisture content

**Fig. 1:**
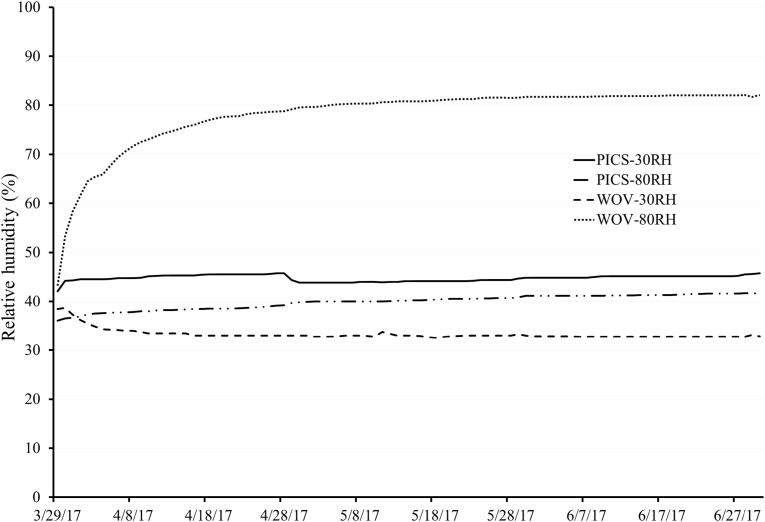
Relative humidity inside polypropylene (PP) woven and Purdue Improved Crop Storage (PICS) bags containing soybean seed and stored at 30% and 80% relative humidities for three months.

Internal r.h. of PICS bags stored at 30 and 80% and PP woven bags stored at 30%, ranged between 33.7 and 46.8%; while that of PP bag stored at 80% r.h. varied between 43.2 and 82.1% (Fig. 1). Moisture content of soybean seed varied among the treatments (F = 84.79; df = 15/56; p < 0.001). No difference was observed in moisture content of soybean seed stored at 30% r.h. in PICS and PP bags and that stored at 80% r.h. in PICS bags (Table 1). However, the moisture content of soybean seed stored in PP bags at 80% r.h. increased by a factor of 2.12 and 2.27 during the first and third months of storage, respectively.

**Table 1:** Moisture content of soybean seed stored in polypropylene (PP) woven and Purdue Improved Crop Storage (PICS) bags at 30% and 80% relative humidities for three months. Values are means ± standard errors. Means in the same column or same raw followed by the same letters are not significantly different at p = 0.05

Treatment*	Initial	Opening time		
		1 month	2 months	3 months
PICS-30RH	6.79 ± 0.25 aA	7.27 ± 0.67 aA	6.70 ± 0.21 aA	7.10 ± 0.29 aA
PICS-80RH	6.61 ± 0.13 aA	6.80 ± 0.40 aA	6.57 ± 0.15 aA	7.53 ± 0.46 aA
PP-30RH	6.80 ± 0.14 aA	7.00 ± 0.35 aA	6.60 ± 0.32 aA	6.30 ± 0.03 aA
PP-80RH	6.50 ± 0.18 aA	13.78 ± .0.57bB	14.50 ± 0.20bB	14.73 ± 0.50bB

*Each treatment had 9 bags at the initial and only 3 bags were open for assessment each month. One sample was collected from each bag.

### 3.3 Seed germination and germination index

Germination rates varied among the treatments (F = 12605.99; df = 15/272; p < 0.001), but significantly decreased for soybean seed stored in PP bags at 80% r.h. (Table 2). Germination of soybean seed stored in PICS bags at 30 and 80%, and in PP bags at 30% were similar, except for PP bags at 30% during the third month when it decreased (Table 2). Decrease in germination rates of soybean stored in PICS bags at 30% and 80%, and PP bags at 30% were 3.2%, 6.0%, and 6.9%, respectively; after three months of storage. However, the germination rate of soybean seed stored in PP bags at 80% r.h. significantly decreased to 11% and less than 1%, respectively, after two and three months of storage. There was a significant negative correlation between grain moisture and soybean seed germination (Pearson correlation = −80.6%, p < 0.001).

**Table 2:** Germination rate of soybean seed stored in polypropylene (PP) woven and Purdue Improved Crop Storage (PICS) bags at 30% and 80% relative humidities for three months. Values are means ± standard errors. Means in the same column or the same raw followed by the same letters are not significantly different at p = 0.05

Treatment*	Initial	Opening time		
		1 month	2 months	3 months
PICS-30RH	99.22 ± 0.31 aA	99.33 ± 0.45 aA	98.33 ± 0.77 aA	96.00 ± 1.39 aA
PICS-80RH	99.33 ± 0.37aAB	100.00 ± 0.00 aA	95.67 ± 0.92aBC	93.33 ± 1.24abC
PP-30RH	98.89 ± 0.52 aA	98.33 ± 1.35 aA	97.00 ± 1.00 aA	92.00 ± 1.91bB
PP-80RH	98.56 ± 0.51 aA	97.33 ± 1.33 aA	10.67 ± 2.05bB	0.67 ± 0.45 cC

*Each treatment had 9 bags at the initial and only 3 bags were open for assessment each month. Four samples (of 25 seed each) were collected from each bag.

The germination index followed the trend of the germination rate and there were significant differences among the treatments (F = 751.56; df = 15/272; p < 0.001). No significant differences were observed between the beginning and the end of the experiment for soybean seed stored in PICS at 30 and 80%. However, germination index for soybean seed stored in PP bags at 30 and 80% r.h. decreased after three months of storage (Table 3).

**Table 3:** Germination index of soybean seed stored in polypropylene (PP) woven and Purdue Improved Crop Storage (PICS) bags at 30% and 80% relative humidities for three months. Values are means ± standard errors. Means in the same column or the same raw followed by the same letters are not significantly different at p = 0.05

Treatment*	Initial	Opening time			
		1 month	2 months	3 months	
PICS-30RH	3.61 ± 0.02 aA	3.65 ± 0.03 aA	3.40 ± 0.04 aB		3.63 ± 0.09 aA
PICS-80RH	3.60 ± 0.01 aA	3.60 ± 0.02bA	3.31 ± 0.03 aB		3.46 ± 0.03 aA
PP-30RH	3.58 ± 0.02 aA	3.60 ± 0.05 aA	3.29 ± 0.06 aB		3.22 ± 0.06bB
PP-80RH	3.55 ± 0.02 aA	3.51 ± .0.06bA	0.31 ± 0.06bB		0.02 ± 0.02 cB

*Each treatment had 9 bags at the initial and only 3 bags were open for assessment each month. Four samples (of 25 seeds each) used for germination were assessed for germination index.

## 4 Discussion

The results show that soybean moisture content and r.h. inside PP bags stored at 80% r.h. doubled during the first month. The r.h. in the PP bags followed the prevailing environmental conditions set in the chamber. This change in r.h. in PP bags stored at 80% r.h. led to increases in the moisture content of soybean seed. Such changes have been observed when soybean seed was stored at a high r.h. (Ali et al., 2020; Chirchir et al., 2017; Nkang and Umoh, 1997). The hygroscopic properties of soybeans are well documented (Ali et al., 2015; Shelar et al., 2008). In PICS bags stored at 80% r.h., however, internal r.h. and soybean seed moisture content remained the same during the three months of storage. Minimal changes in r.h. have been observed inside PICS bags during grain storage (Bakoye et al., 2020; Williams et al., 2017) and raffia bags coated with polyethylene liners during seed storage (Coradi et al., 2020a; 2020b).

The germination rate of soybean seed kept in the PP bag at 80% r.h. was significantly affected by the moisture content and decreased by 87.89% and 97.9% after two and three months of storage, respectively. Similar results were obtained when soybean seed was stored at 80% r.h. for two or three months using porous materials such as cloth bags (Ali et al., 2015; Miah et al., 2006). High moisture levels facilitate the development of mycoflora which contributes to the deterioration of soybean seed quality during storage (Shelar et al., 2008). The absorption of water by soybeans triggers physiological changes leading to the reduction of seed viability (Ndimande et al., 1981). These physico-enzymatic processes may explain the drastic drop in the germination rates observed in soybean seed stored in PP bags at 80% r.h. for two or more months (Ali et al., 2015; Miah et al., 2006; Nkang and Umoh, 1997).

PICS bags were effective in maintaining soybean seed quality at high r.h. Storage in hermetic laminated packaging, glass jars, or raffia bags coated with polyethylene liners has shown similar results (Ali et al., 2015, 2020). Hermetic containers can maintain low maize and soybean seed moisture content; hence preserve high germination rates (Afzal et al., 2017; Ali et al., 2020). This research showed that the lower moisture content was correlated with a high soybean germination rate. Further, PICS and PP bags had similar oxygen levels which may be explained by the lack of insects in any of the treatments. This is not uncommon as high oxygen levels have been observed in PICS bags during storage of non-infested grain (Williams et al., 2017). Though seeds are living organisms, their oxygen requirement is minimal. This may explain, in part, why the quality of seed is preserved during storage under hermetic conditions. The germination index also demonstrates the high vigor of soybean seed stored in PICS bags.

## 5 Conclusion

This study demonstrated that PICS bags can maintain soybeans seed quality at high r.h. for up to three months. Germination was minimally affected when soybean seed was stored at 80% r.h. in PICS bags compared to PP woven bags. Using hermetic bags (e.g., PICS bags) to preserve soybean seed quality would be a viable and affordable solution for millions of farmers who are already using the same technology to store grain. This will help farmers access good planting materials; therefore, contribute to better crop productivity. Also, seed producers/traders can use hermetic bags to preserve the quality of soybean seed supplied to farmers. These results suggest that PICS bags could potentially be used to preserve the quality of foodstuffs (e.g., dried vegetables and fruits) that are sensitive to high r.h. during storage; but more research is needed.

## Author contributions

Conceptualization, DB and I.B.B.; methodology, DB and I.B.B.; Software, DB and I.B.B.; validation, DB and I.B.B.; formal analysis, DB and I.B.B.; investigation, DB; resources, D.B.; data curation, DB and I.B.B.; writing—original draft preparation, DB and I.B.B.; writing—review and editing, DB and I.B.B.; visualization, DB and I.B.B.; supervision, D.B.; project administration, D.B.; funding acquisition, D.B, All authors have read and agreed to the published version of the manuscript.

## Declaration of competing interest

The authors declare the following financial interests/personal relationships, which may be considered as potential competing interests: Dieudonne Baributsa is a co-founder of PICS Global Inc., a social enterprise that commercializes postharvest technologies (including PICS bags) to small-holder farmers around the world and hence declares a conflict of interest. Ibrahim Boukary Baoua declares no conflict of interest. The funder (10.13039/100000865Bill and Melinda Gates Foundation) had no role in the design of the study; in the collection, analyses, or interpretation of data; in the writing of the manuscript, or in the decision to publish the results.
